# Fabry's disease: An ultrastructural study of nerve biopsy

**DOI:** 10.4103/0972-2327.42939

**Published:** 2008

**Authors:** N. Gayathri, T. C. Yasha, Makarand Kanjalkar, Santosh Agarwal, B. K. Chandrashekar Sagar, Vani Santosh, S. K. Shankar

**Affiliations:** Department of Neuropathology, National Institute of Mental Health and Neurosciences, Bangalore, India; 1 Manik Hospital and Research Center, Garkheda, India; 2 Sushila Netralaya, Bansilal Nagar, Aurangabad, India

**Keywords:** Angiokeratomas, Fabry's disease, lamellated inclusions

## Abstract

Fabry's disease, an X linked recessive disorder caused by the deficiency of α-galactosidase A (α-gal A), leads to progressive accumulation of glycosphingolipids. We report this rare disease in a 19-year-old boy who presented with angiokeratomas, paresthesia and corneal opacities, and nerve biopsy revealed by electron microscopy lamellated inclusions in the smooth muscle, perineurial and endothelial cells characteristic of Fabry's disease.

## Introduction

Fabry's disease, an X linked recessive disorder, caused by the deficiency of α-galactosidase A (α-gal A), causes progressive accumulation of glycosphingolipids in the visceral tissue and vascular endothelium, leading to multisystem manifestations. The disease gene is mapped to Xq21-33 and Xq22, and more than 200 mutations have been identified.[1] Onset is usually in childhood, with severe pain in the extremities, vesicular cutaneous lesions and corneal and lenticular opacities. The disease is rare with an estimated incidence of one in 40,000 males. Review of Indian literature reveals five reported cases, which mainly describe clinical features with dermatological involvement. There is one report on pathological changes in the skin biopsy.[2]

For the first time, we report the detailed ultrastructural features of nerve biopsy in Fabry's disease.

## Case Report

A 19-year-old boy, the only son of non-consanguinous parents, presented with severe paresthesia in both hands and feet, occurring on and off since eight years. He had continuous burning sensation for many weeks, causing disturbed sleep. He had to discontinue schooling due to this. He complained of pain which was exaggerated with fever. When the temperature was under control, his symptoms improved. His pain responded excellently to Gabapentin. There was no family history of this condition.

**Figure 1:**
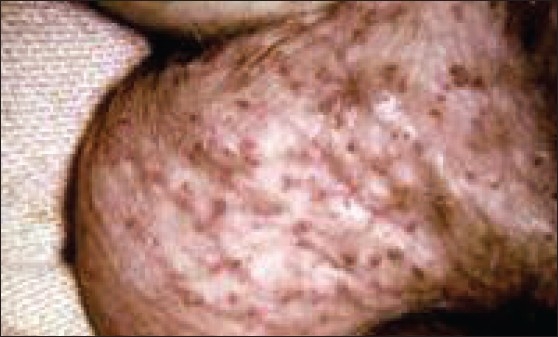
Multiple cherry red colored raised angiokeratomas noted over the trunk

On examination, multiple cherry red colored raised lesions, suggestive of angiokeratomas, were noted over the trunk [Figure 1]. Ophthalmologic evaluation revealed whorl-like corneal opacities and conjunctival vessel abnormalities. On neurological examination, it was found that the power in all four limbs was normal, with intact deep tendon reflexes, normal touch, pin prick, joint position and vibration sensations, but decreased temperature sense, distally in the lower limbs. There was no nerve thickening. Motor nerve conduction in the upper and lower limbs, including F-waves, was normal. Sensory nerve conduction was normal in the upper limbs, while sural Sensory Nerve Action Potential (SNAP) were low bilaterally. Investigations revealed normal hemogram; HIV was non-reactive. Urine analysis showed traces of albumin. Kidney function tests, cardiac tests, and ultrasound abdomen tests were unremarkable.

A clinical diagnosis of Fabry's disease was considered. Sural nerve biopsy submitted for diagnosis was processed for light and electron microscopic studies. For light microscopy, paraffin sections were stained with Haematoxylin eosin (HE), Masson's trichrome (MAT) for collagen, and Kultshitzky-Pal for myelin. Tiny pieces of nerve post-fixed in osmium tetroxide were embedded in araldite, for electron microscopy. 1 µ thick plastic sections were stained with methelene blue-azure II for light microscopy, while ultra thin sections contrasted with uranyl acetate and lead citrate were scanned under JEOL 100 CX electron microscope at 60 KV.

### Pathological features

*Light microscopy:* HE and MAT stained paraffin sections were unremarkable, except for one focus of endoneurial perivascular lymphocytic cuffing [Figure 2A]. 1 µ plastic section stained with methelene blue-azure II revealed abundant osmiophilic, granular deposits of varying sizes in perineurial cells, and endothelial and smooth muscle cells of the blood vessel [Figure 2B]. Schwann cells did not contain similar material. There was chronic axonopathy, with mild depletion of small and large diameter fibers. A few regenerating clusters were noted.

**Figure 2A:**
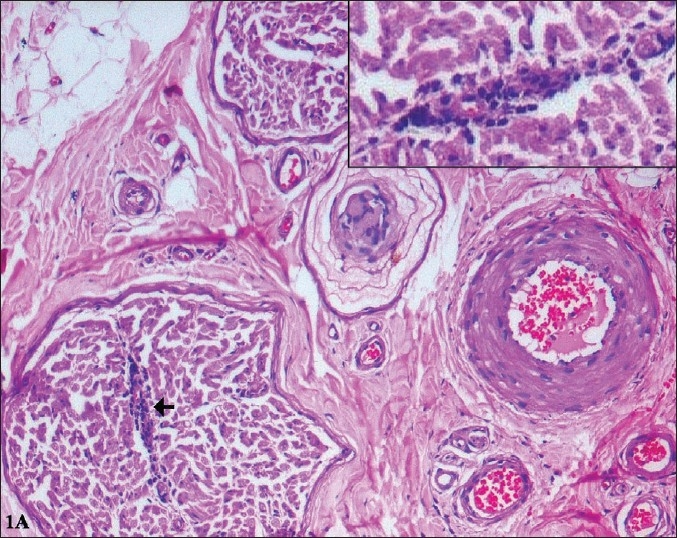
Perivascular inflammation is seen around a small arteriole within the endoneurium (arrow) (H&E, ×120) Inset: Higher magnification showing the lymphocytic cuffing. (H&E, ×240)

**Figure 2B:**
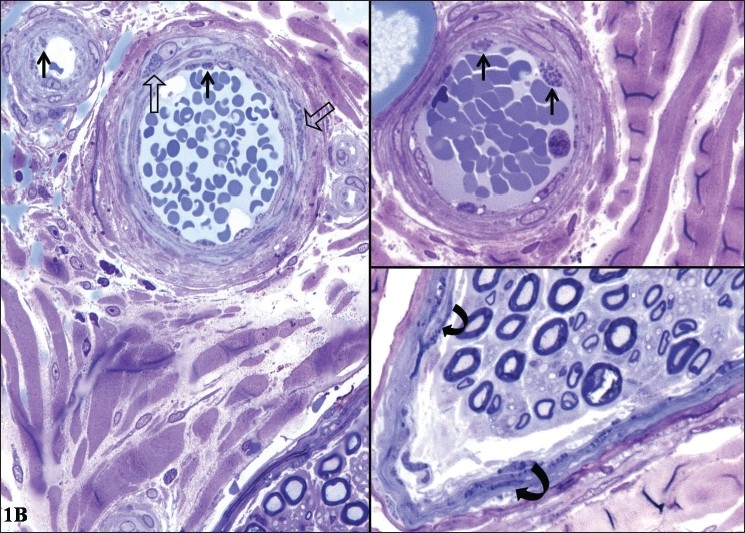
One micron thick, semithin sections of plastic embedded nerve tissue, stained with methylene blue-azure II show multiple, dark osmiophilic granules within the endothelial cells (closed arrows) and smooth muscle cells (open arrows) of the epineurial blood vessels (A, B). (×350)

**Figure 3:**
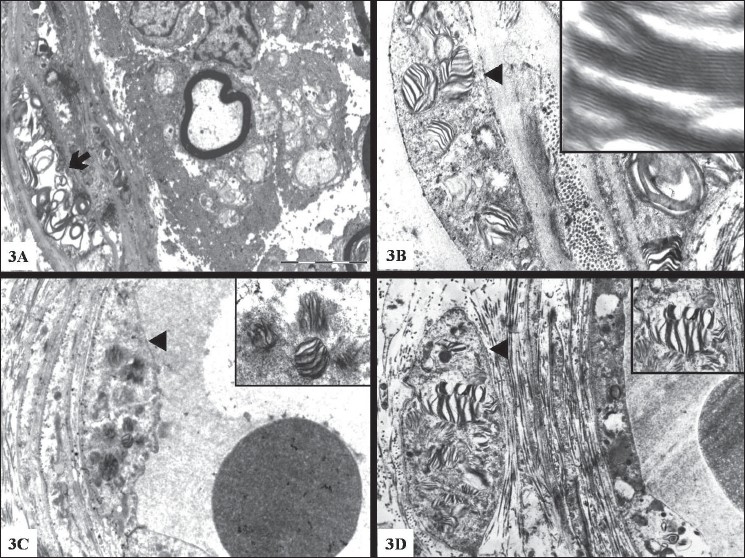
Electron micrograph of a portion of nerve showing concentric rings (arrow) (3A) and Zebra bodies (arrow head) (3B) in the perineurial cells. Note inclusions in the endothelial cells (3C) and smooth muscle (3D) of blood vessel. 3B: High magnification (×1,00,00) showing 3 nm lamellated pattern Magnification: 3A & 3C ×11,500 3B & 3D 23,000

*Electron microscopy:* Abundant osmiophilic inclusions were noted in the vascular endothelial and smooth muscle cells, pericytes, fibroblasts and perineurial cells [Figure 3]. The inclusions were round to oval and a majority was membrane bound. They were predominantly lamellated, forming Zebra bodies [Figure 3B]. A few were amorphous electron dense structures. In addition, regular arrays of concentric rings [Figure 3A] were also noted. Higher magnification of the lamella resolved 3nm periodicity [Inset Figure 3B]. Similar inclusions were not noted in the Schwann cells or the axons. Nucleus, Golgi and mitochondria were unremarkable. There was focal depletion of small and large diameter fibers, with increased endoneurial collagen. The unmyelinated fiber density was normal. In an adult with angiokeratomas, corneal and lenticular opacities and peripheral neuropathy, the ultrastructural examination of the nerve biopsy revealing lamellated osmiophilic inclusions in the perineurial cells and vascular endothelial cells, a diagnosis of Fabry's disease, accumulation due to α-galactosidase deficiency was considered.

## Discussion

Fabry's disease, also known as angiokeratoma corporis diffusum due to the presence of cutaneous angiokeratomas, is an X linked disease, caused by the deficiency of α-galactosidase A (α-gal A), leading to accumulation of glycosphingolipids, particularly globotriasylceramide (Gb3), predominantly in vascular endothelial and smooth muscle cells. Painful paresthesias, angiokeratomas, lenticular opacities and hypohydrosis characterize the disease. The widespread vascular pathology explains the multisystem involvement in typical cases affecting renal, cardiac and central nervous systems, leading to morbidity and mortality. Detection of enzyme deficiency and gene mutations have helped to recognize atypical manifestations with single system involvement, like isolated renal pathology, hypertrophic cardiomyopathy, or stroke due to multiple microinfarcts. Heterozygote female carriers may also manifest with the disease, although of less severe intensity

In some patients, the presence of fever, elevated erythrocyte sedimentation rate and rheumatic manifestations have led to an initial misdiagnosis of connective tissue disease,[3,4] which has been further compounded by a high association with autoantibodies.[5] There is no satisfactory explanation for these observations, although some authors suggest that lipid storage stimulates the production of autoantobodies.[6,7] Inflammation in the nerve biopsy has been mentioned in two reports.[8,9] In one,[8] it was a nonvasculitic epineurial perivascular inflammation, a finding that may be a nonspecific occurrence.[10] In the present case, there was significant endoneurial perivascular inflammation, although only around one vessel. The significance of this finding is presently unclear.

The patient was otherwise typical in presentation and was a young male adult with angiokeratomas, parestheisia and corneal opacities characteristic of Fabry's disease, with normal renal and cardiac functions. Ultrastructurally, osmiophilic lamellated (Zebra bodies) and amorphous inclusions suggesting lipid storage material was noted in the vascular endothelial and smooth muscle cells, and in the perineurial cells and fibroblasts. The inclusions were within a single membrane bound organellae, suggesting their presence in the lysosomes. The presence of storage material within lysosomes could be due to the deficiency of α-gal A within the lysosomes, causing accumulation rather than degradation. Zebra bodies in the peripheral nerve are seen in other conditions like metachromatic leucodystrophy (MLD). The inclusions in MLD are noted in Schwann cells of myelinated and unmyelinated fibers, in addition to their presence in the endothelial cells and fibroblasts, unlike its absence in Schwann cells in Fabry's disease.

The cause for pain has been attributed to the involvement of dorsal root ganglion cells and neurons of the autonomic system, with Gb3 accumulation. With increase in age, Gb3 is known to progressively accumulate throughout the body, leading to multisystem involvement. Early diagnosis and treatment by enzyme replacement therapy is essential to prevent multisystem involvement and to prevent stroke, myocardial and renal failures.[11,12]

Relevant enzyme assays could not be carried out in our case. Nevertheless, we emphasize the importance of examination of nerve biopsy in establishing the diagnosis. Examination of semi-thin and ultra-thin sections of the nerve has a definite role in offering an accurate diagnosis in Fabry's disease.

## References

[1] Desnik RJ, Ioannou YA, Eng MC, Scriver CR, Beaudet AL, Shy WS, Valle D (2001). α-galactose A deficiency: Fabry disease. The metabolic and molecular bases of Inherited diseases.

[2] Amin SS, Jahseen M, Ahamed Z, Zaheer MS, Perwin N (2004). Angiokeratoma corporis diffusum (Fabry's disease). JIACM.

[3] Rahman P, Gladman DD, Wither J, Silver MD (1998). Coexistence of Fabry's disease and systemic lupus erythematosus. Clin Exp Rheumatol.

[4] Paira SO, Roverano S, Iribas JL, Barceló HA (1992). Joint manifestations of Fabry's disease. Clin Rheumatol.

[5] Martinez P, Aggio M, Rozenfeld P (2007). High incidence of autoantibodies in Fabry disease patients. J Inherit Metab Dis.

[6] Rosenmann E, Kobrin I, Cohen T (1983). Kidney involvement in systemic lupus erythematosus and Fabry's disease. Nephron.

[7] Arias Martínez N, Barbado Hernández FJ, Pérez Martí;n G, Pérez de Ayala C, Casal Esteban V, Vázquez Rodrí;guez JJ (2003). Fabry's disease associated with rheumatoid arthritis. Multisystemic crossroads. An Med Interna.

[8] Lacomis D, Roeske-Anderson L, Mathie L (2005). Neuropathy and Fabry's disease. Muscle Nerve.

[9] Toyooka A, Said G (1997). Nerve biopsy findings in hemizygous and heterozygous patients with Fabry's disease. J Neurol.

[10] Gyl M, Bilbao JM (1995). Biopsy diagnosis of Peripheral Neuropathy.

[11] Schiffmann R (2006). Neuropathy and Fabry disease: Pathogenesis and enzyme replacement therapy. Acta Neurol (Belq).

[12] Eng CM, Guffon N, Wilcox WR, Germain DP, Lee P, Waldek S (2001). Safety and efficacy of recombinant human alpha-galactosidase A replacement therapy in Fabrys disease. N Engl J Med.

